# Supplementary material for: Molecular basis for assembly and activation of the Hook3 − KIF1C complex-dependent transport machinery
Source: EMBO Rep. 2025 May 1;26(11):2945–66. doi: 10.1038/s44319-025-00458-w (PMC12152161; doi:10.1038/s44319-025-00458-w)
Supplement: Supplementary file 1 — Table EV1 [file 44319_2025_458_MOESM1_ESM.docx]

**Table EV1. Data collection and structure refinement statistics**

|  | **Hook3(553–624)**− **KIF1C(714–809)** | **SeMet-substituted Hook3(553–624)** |
| --- | --- | --- |
| **PDB code** | **9KO8** | **9KNS** |
| **Data Collection** |  |  |
| Space group | *P*2_1_2_1_2 | *P*6_1_22 |
| Unit cell dimensions |  |  |
| a, b, c (Å) | 54.6, 166.9, 39.1 | 78.7, 78.7, 180.0 |
| α, β, γ (^o^) | 90, 90, 90 | 90, 90, 120 |
| Resolution (Å) | 50.0−2.8 (2.85−2.80) | 50.0−2.7 (2.75−2.70) |
| *R*_sym_^b^ (%) | 11.7 (26.3) | 7.6 (26.4) |
| *I*/σ(*I*) | 10.3 (2.0) | 26.5 (3.5) |
| Completeness (%) | 93.6 (84.5) | 99.0 (99.1) |
| Redundancy | 4.6 | 9.8 |
| **Refinement** |  |  |
| Resolution (Å) | 50.0−3.0 | 50.0−2.7 |
| Number of reflections | 7239 | 9641 |
| *R*_work_^c^/*R*_free_ (%) | 24.1/29.1 | 23.0/26.8 |
| Number of atoms |  |  |
| Protein | 2145 | 1241 |
| Water | 27 | 29 |
| RMSD |  |  |
| Bond lengths (Å) | 0.010 | 0.010 |
| Bond angles (^o^) | 1.201 | 1.100 |
| Ramachandran plot (%) |  |  |
| Most favored region | 94.4 | 98.6 |
| Additionally allowed region | 5.6 | 1.4 |
| Average B-values (Å^2^) |  |  |
| Protein | 43.5 | 51.9 |
| Water | 34.7 | 37.5 |

^a^The numbers in parentheses are statistics from the shell with the highest resolution.

^b^*R*_sym_ = Σ |*I*_obs_ - *I*_avg_| / *I*_obs_, where *I*_obs_ is the observed intensity of individual reflection and *I*_avg_ is the average across symmetry equivalents.

^c^*R*_work_ = Σ ||*F*_o_| - |*F*_c_|| / Σ |*F*_o_|, where |*F*_o_| and |*F*_c_| are the observed and calculated structure factor amplitudes, respectively. *R*_free_ was calculated with 10.0% of the data.
