# Supplementary figures and images for: Molecular basis for assembly and activation of the Hook3 − KIF1C complex-dependent transport machinery
Source: EMBO Rep. 2025 May 1;26(11):2945–66. doi: 10.1038/s44319-025-00458-w (PMC12152161; doi:10.1038/s44319-025-00458-w)

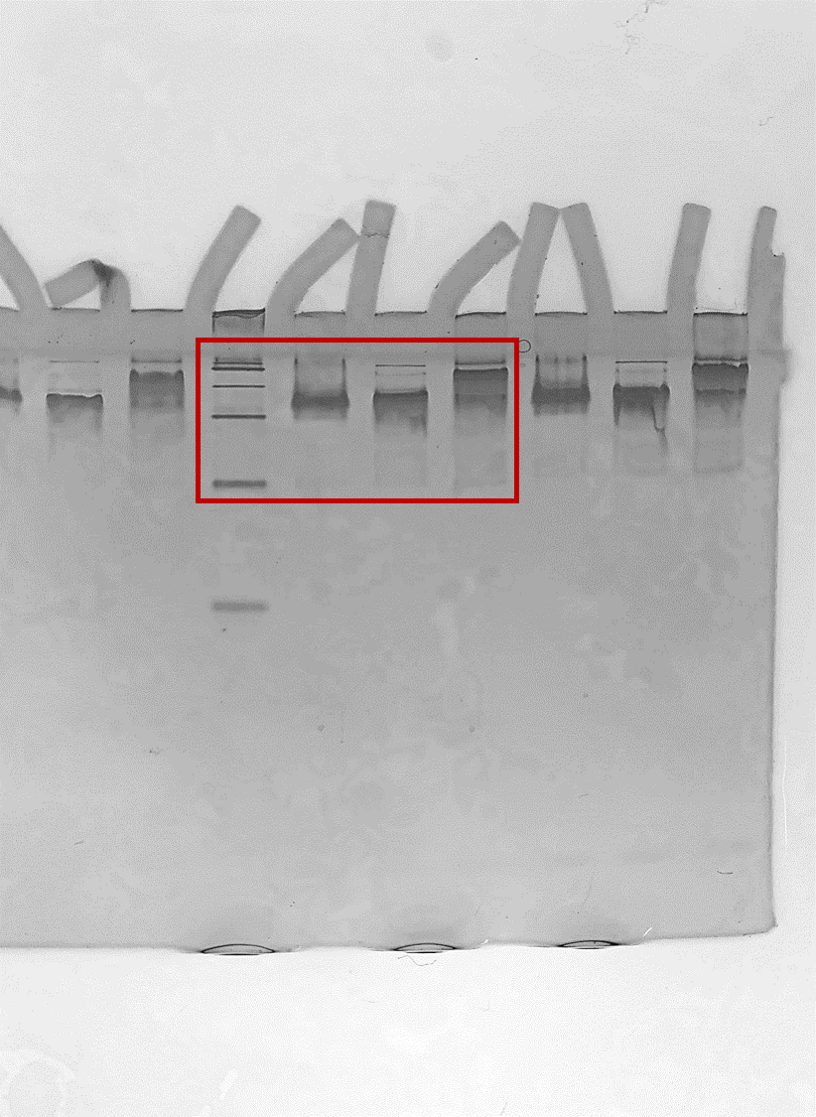

Supplement: Supplementary file 7 — Source data Fig. 1 [file 44319_2025_458_MOESM7_ESM.zip › 1B/1B_Highlighting_nativegel_middle.tif]

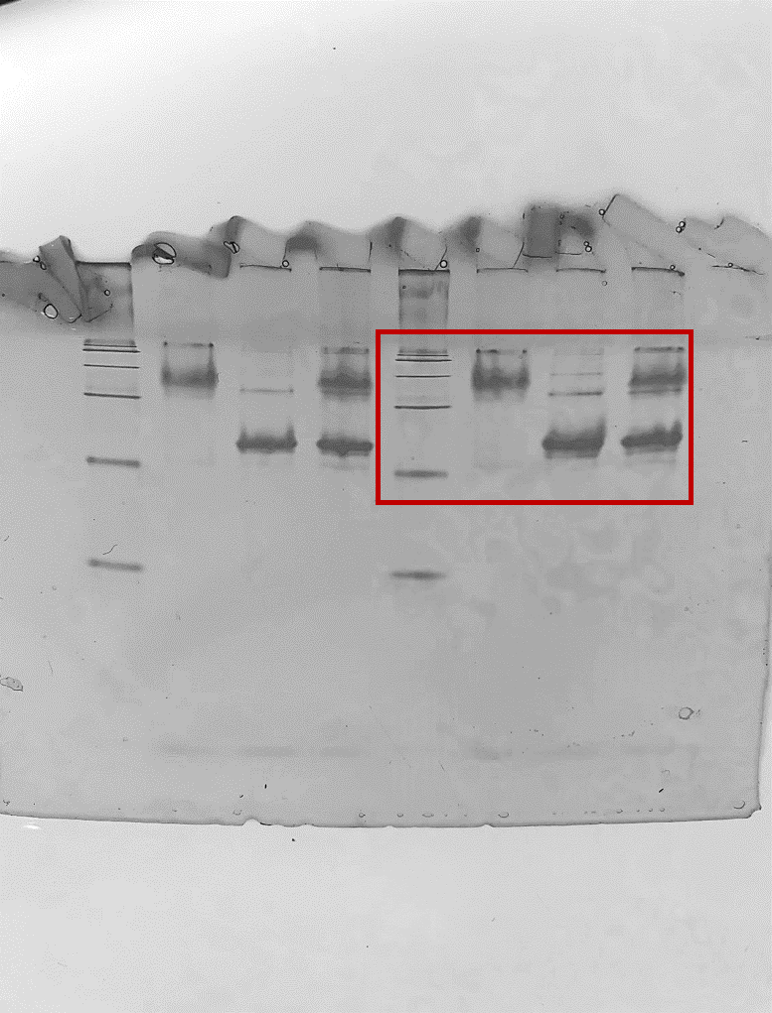

Supplement: Supplementary file 7 — Source data Fig. 1 [file 44319_2025_458_MOESM7_ESM.zip › 1B/1B_Highlighting_nativegel_right.tif]

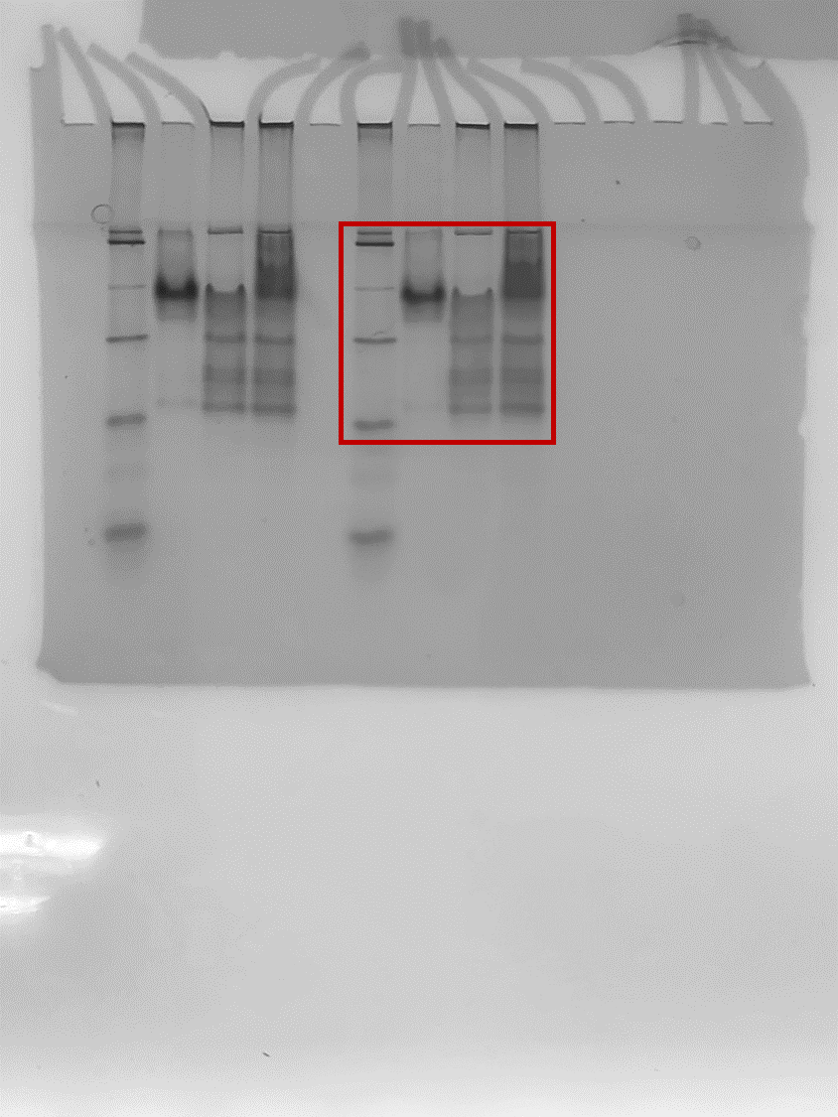

Supplement: Supplementary file 7 — Source data Fig. 1 [file 44319_2025_458_MOESM7_ESM.zip › 1B/1B_Highligting_nativegel_left.tif]

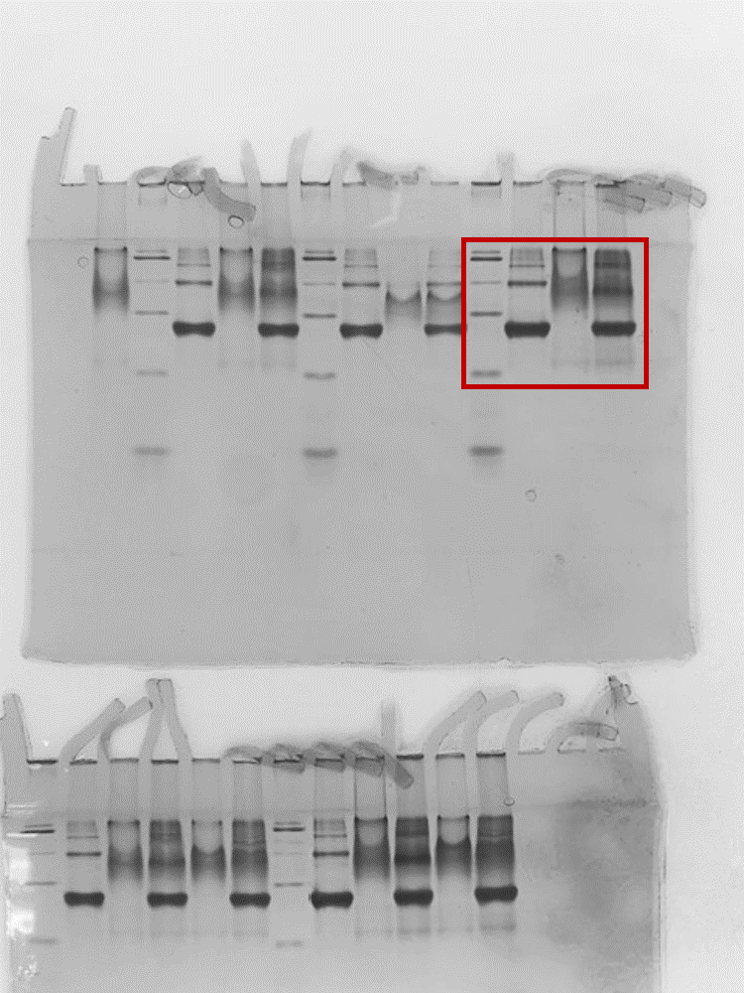

Supplement: Supplementary file 7 — Source data Fig. 1 [file 44319_2025_458_MOESM7_ESM.zip › 1C/1C_Highlighting_native gel_left.tif]

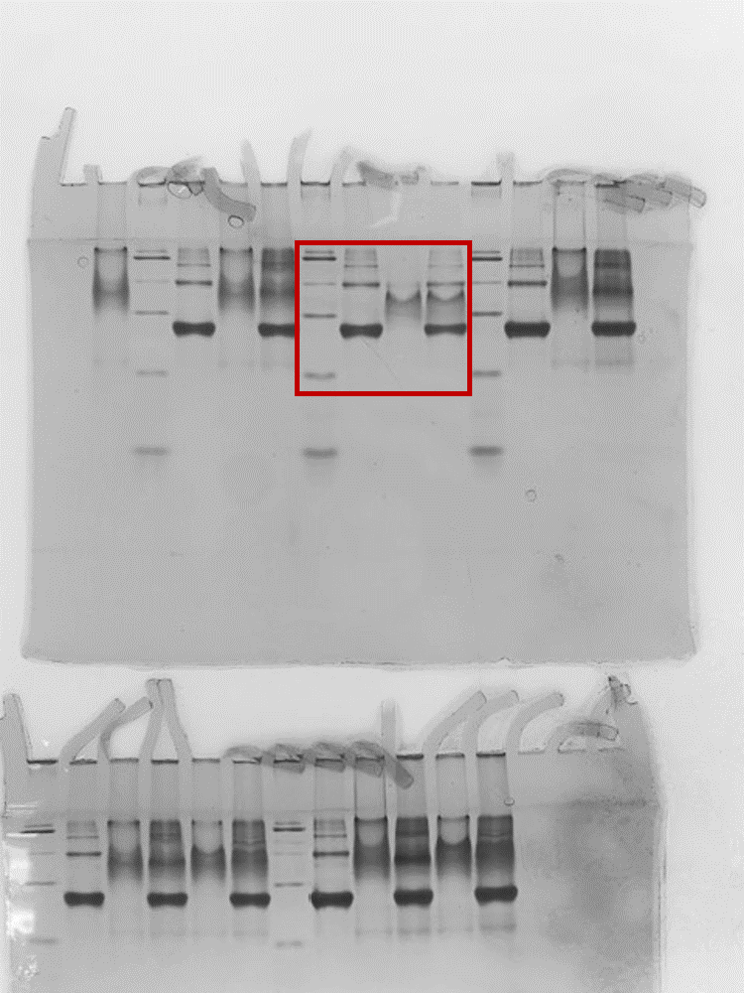

Supplement: Supplementary file 7 — Source data Fig. 1 [file 44319_2025_458_MOESM7_ESM.zip › 1C/1C_Highlighting_native gel_right.tif]

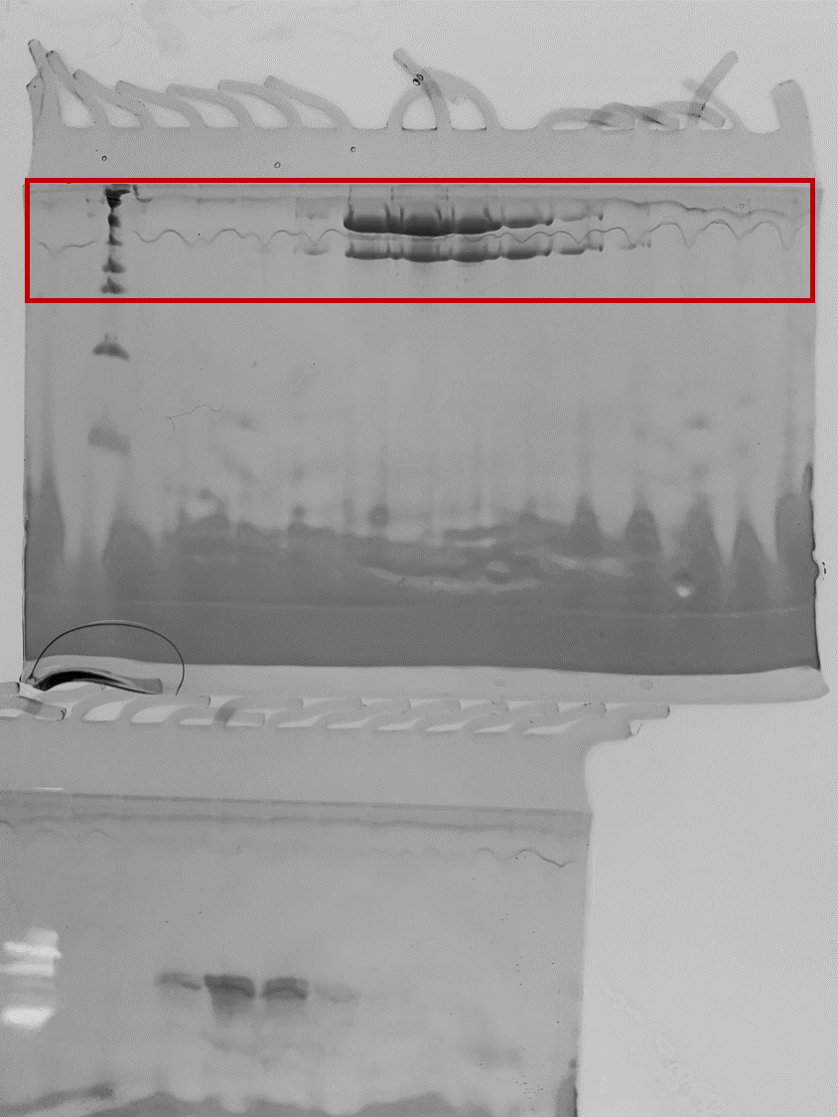

Supplement: Supplementary file 7 — Source data Fig. 1 [file 44319_2025_458_MOESM7_ESM.zip › 1E/1E_Highlighting_size-exclusion chromatography_panel 1.tif]

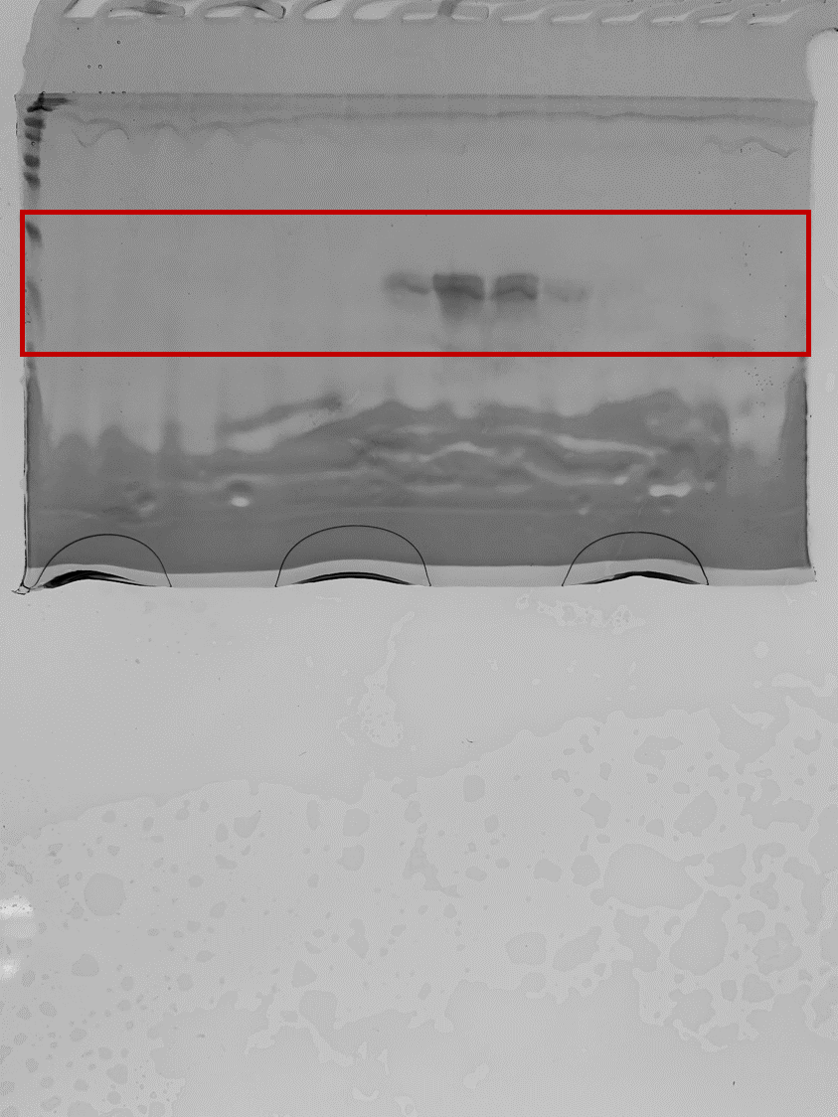

Supplement: Supplementary file 7 — Source data Fig. 1 [file 44319_2025_458_MOESM7_ESM.zip › 1E/1E_Highlighting_size-exclusion chromatography_panel 2.tif]

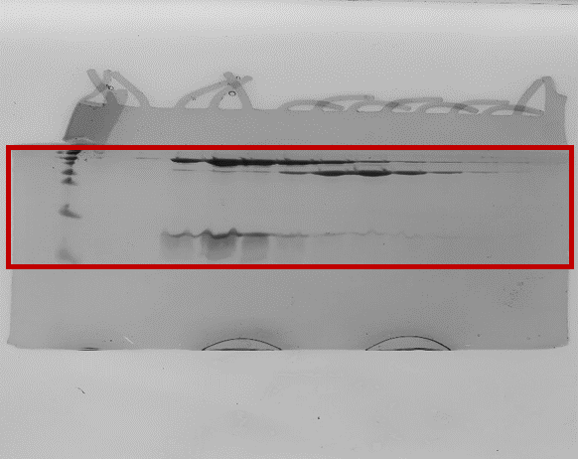

Supplement: Supplementary file 7 — Source data Fig. 1 [file 44319_2025_458_MOESM7_ESM.zip › 1E/1E_Highlighting_size-exclusion chromatography_panel 3.tif]

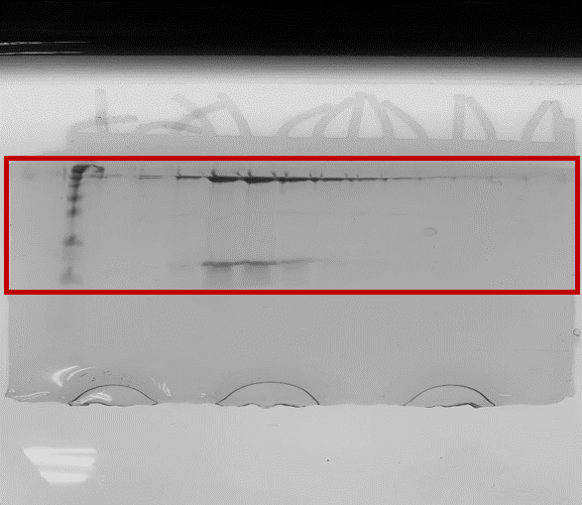

Supplement: Supplementary file 7 — Source data Fig. 1 [file 44319_2025_458_MOESM7_ESM.zip › 1E/1E_Highlighting_size-exclusion chromatography_panel 4.tif]

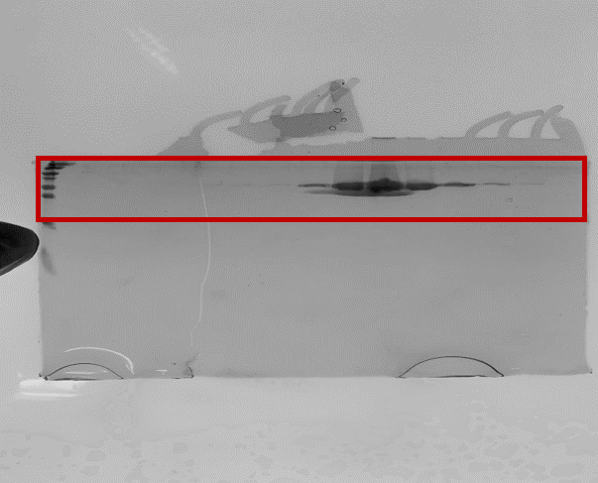

Supplement: Supplementary file 7 — Source data Fig. 1 [file 44319_2025_458_MOESM7_ESM.zip › 1E/1E_Highlighting_size-exclusion chromatography_panel 5.tif]

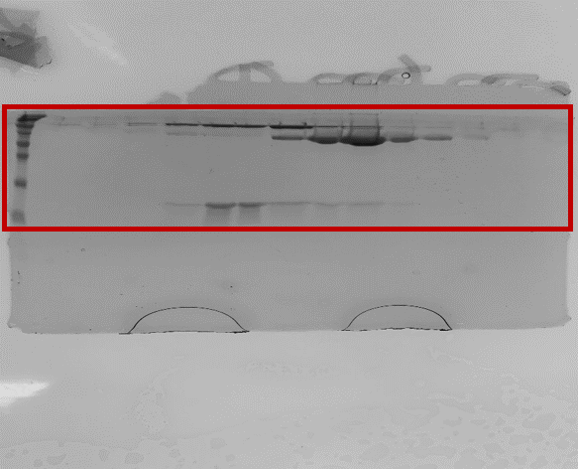

Supplement: Supplementary file 7 — Source data Fig. 1 [file 44319_2025_458_MOESM7_ESM.zip › 1E/1E_Highlighting_size-exclusion chromatography_panel 6.tif]

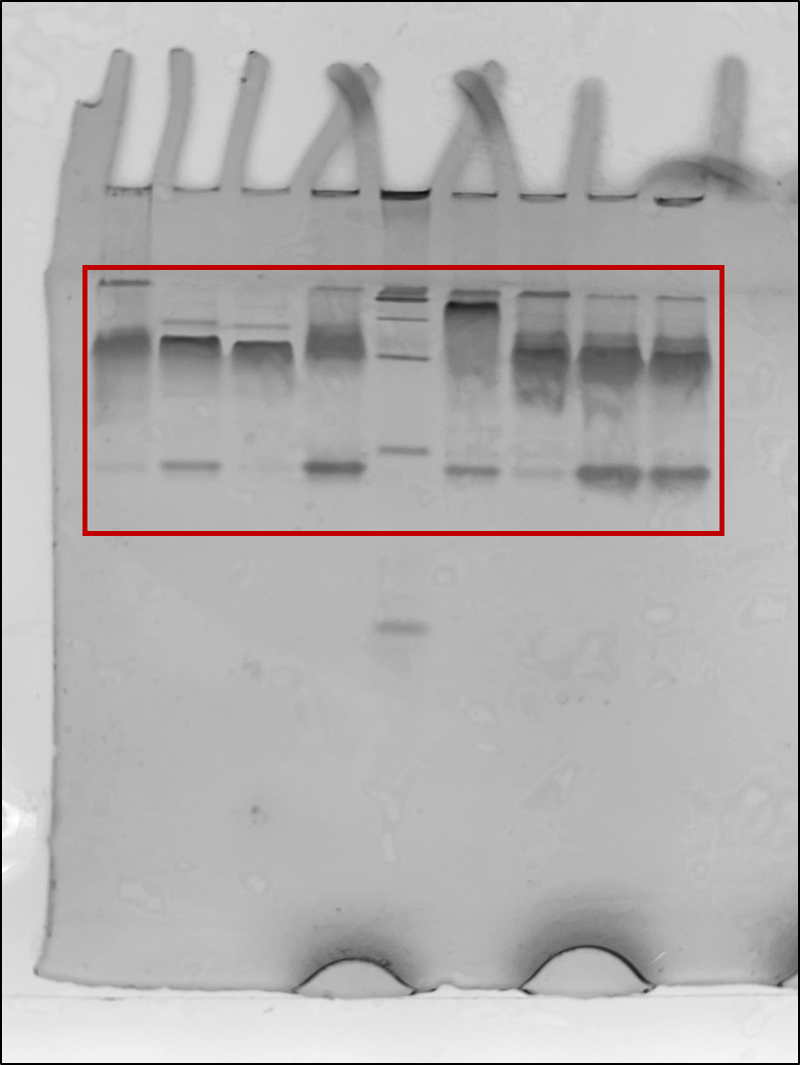

Supplement: Supplementary file 8 — Source data Fig. 3 [file 44319_2025_458_MOESM8_ESM.zip › A/3A_Highlighting_native gel.tif]

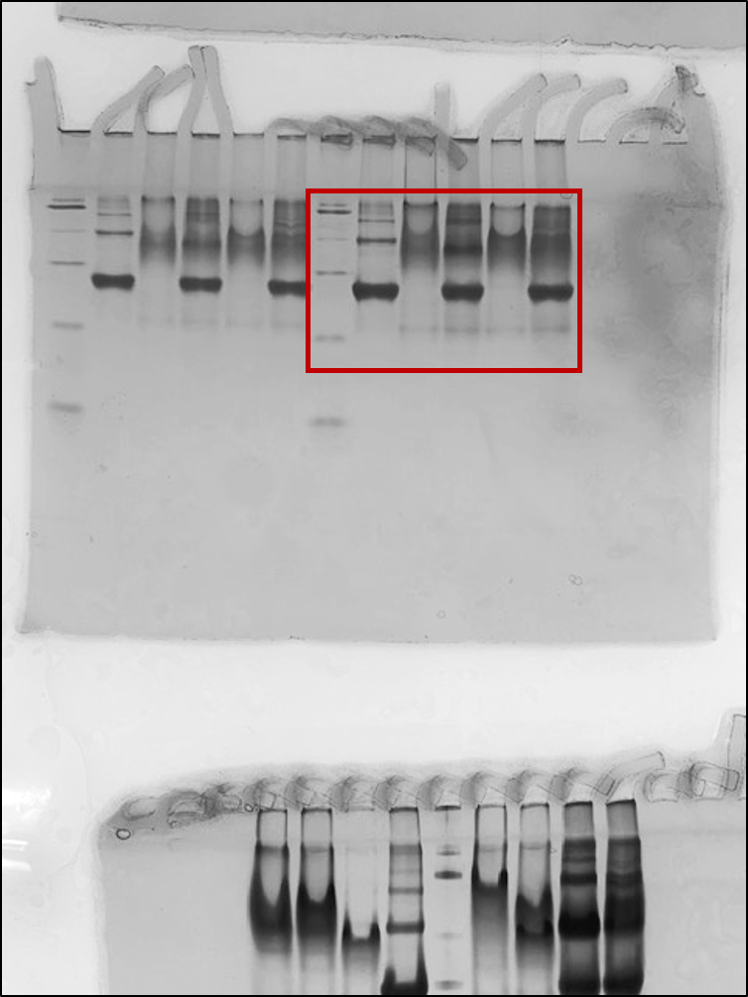

Supplement: Supplementary file 8 — Source data Fig. 3 [file 44319_2025_458_MOESM8_ESM.zip › B/3B_Highlighting_native gel.tif]

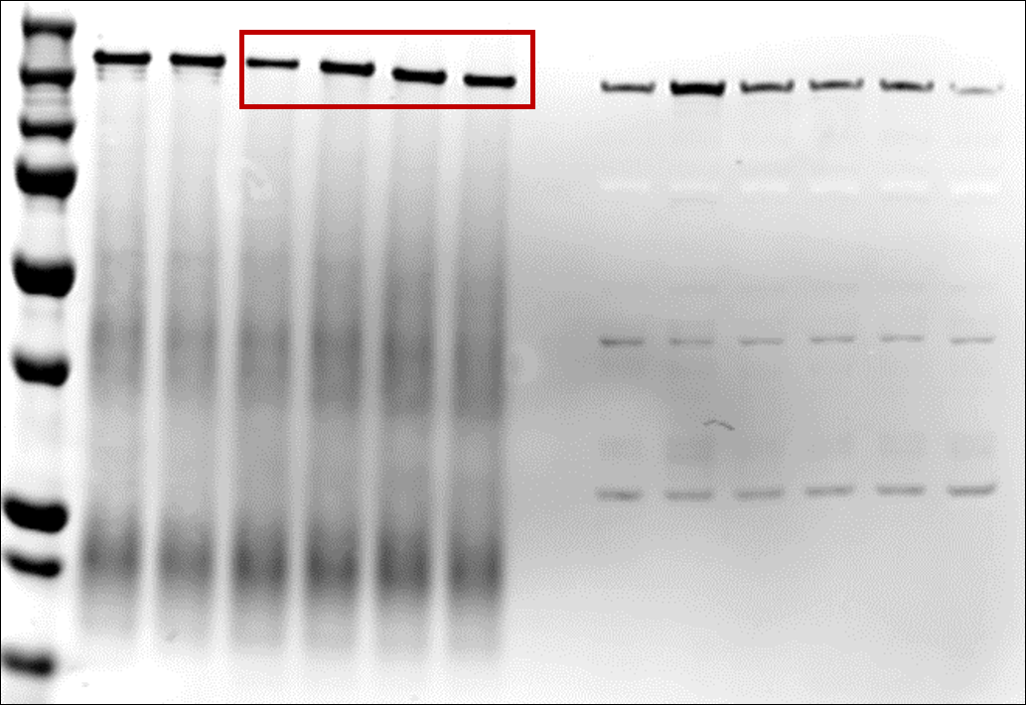

Supplement: Supplementary file 8 — Source data Fig. 3 [file 44319_2025_458_MOESM8_ESM.zip › C/3C_Highlighting_IP_Flag_western blot_Flag.tif]

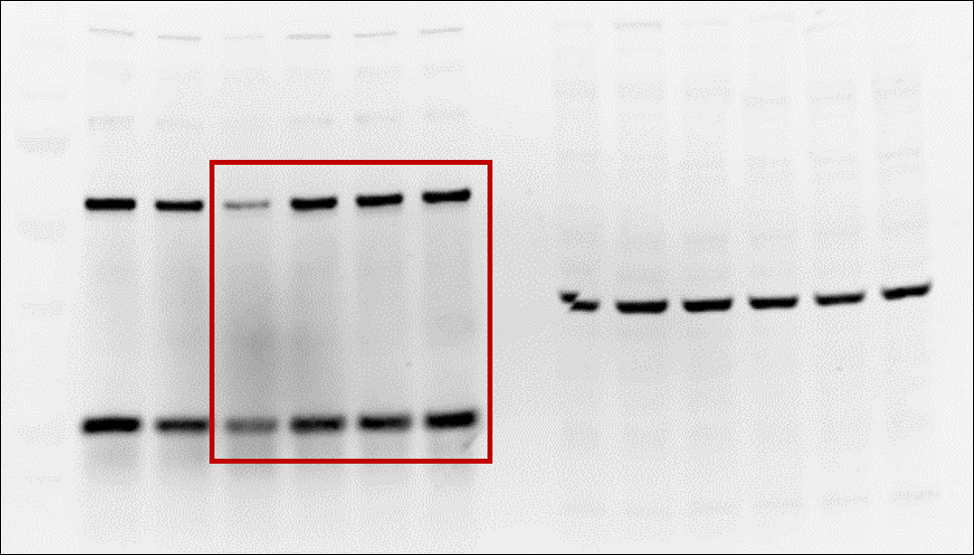

Supplement: Supplementary file 8 — Source data Fig. 3 [file 44319_2025_458_MOESM8_ESM.zip › C/3C_Highlighting_IP_Flag_western blot_GAPDH.tif]

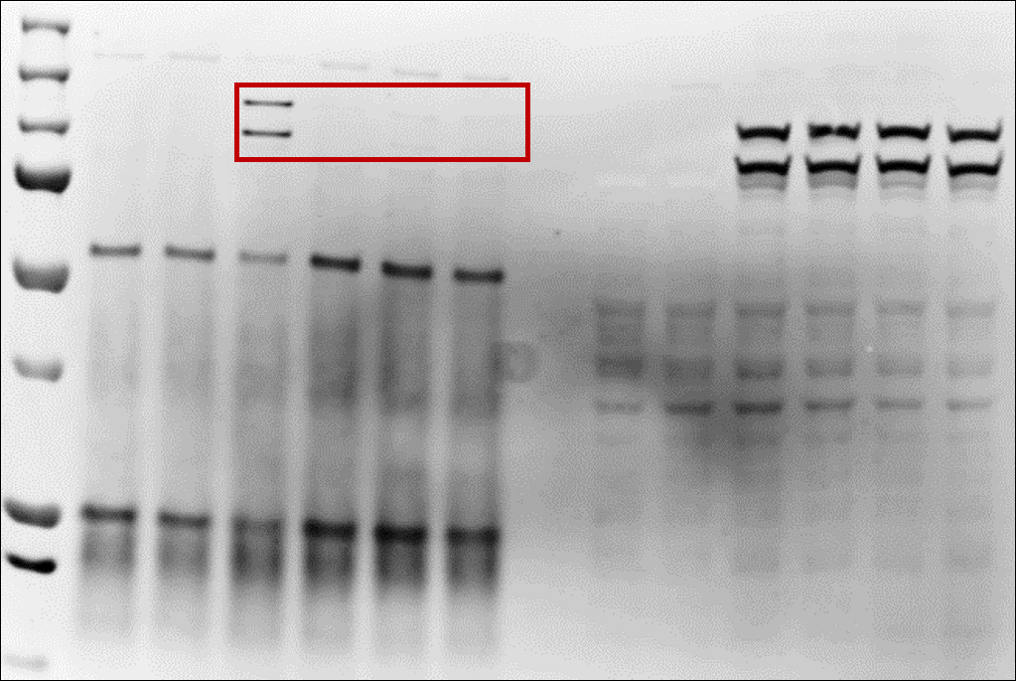

Supplement: Supplementary file 8 — Source data Fig. 3 [file 44319_2025_458_MOESM8_ESM.zip › C/3C_Highlighting_IP_Flag_western blot_Myc.tif]

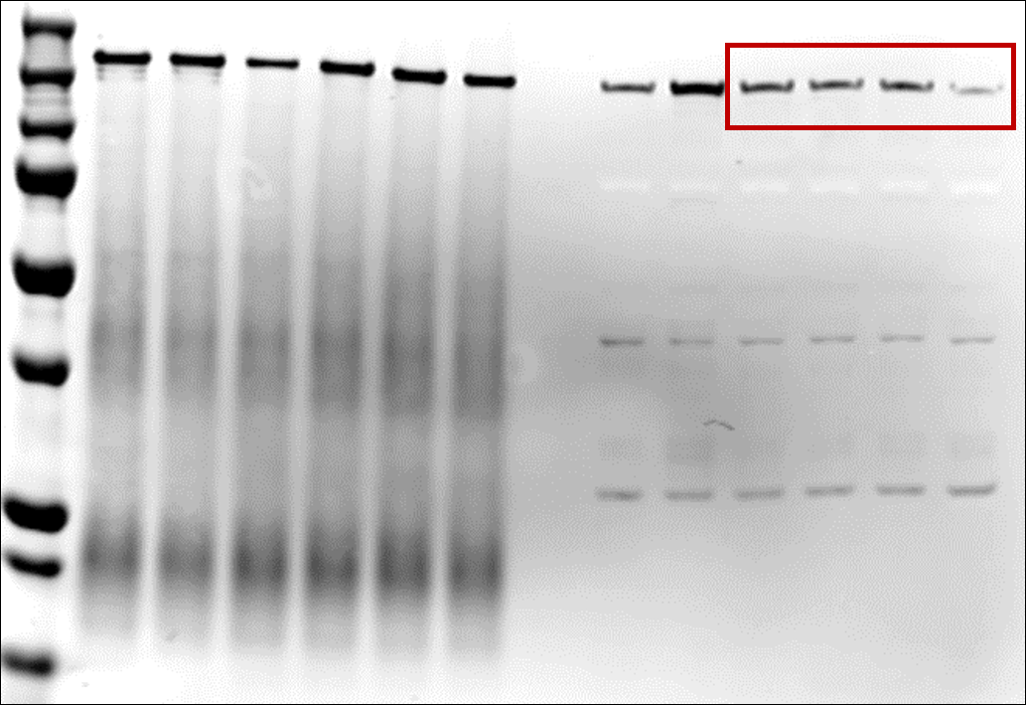

Supplement: Supplementary file 8 — Source data Fig. 3 [file 44319_2025_458_MOESM8_ESM.zip › C/3C_Highlighting_WCL_western blot_Flag.tif]

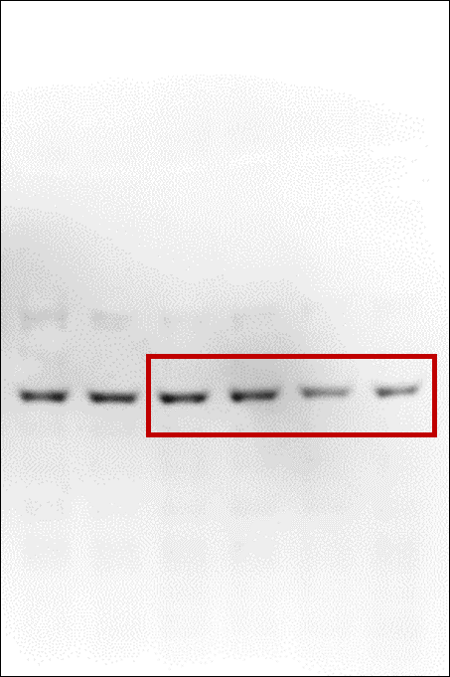

Supplement: Supplementary file 8 — Source data Fig. 3 [file 44319_2025_458_MOESM8_ESM.zip › C/3C_Highlighting_WCL_western blot_GAPDH.tif]

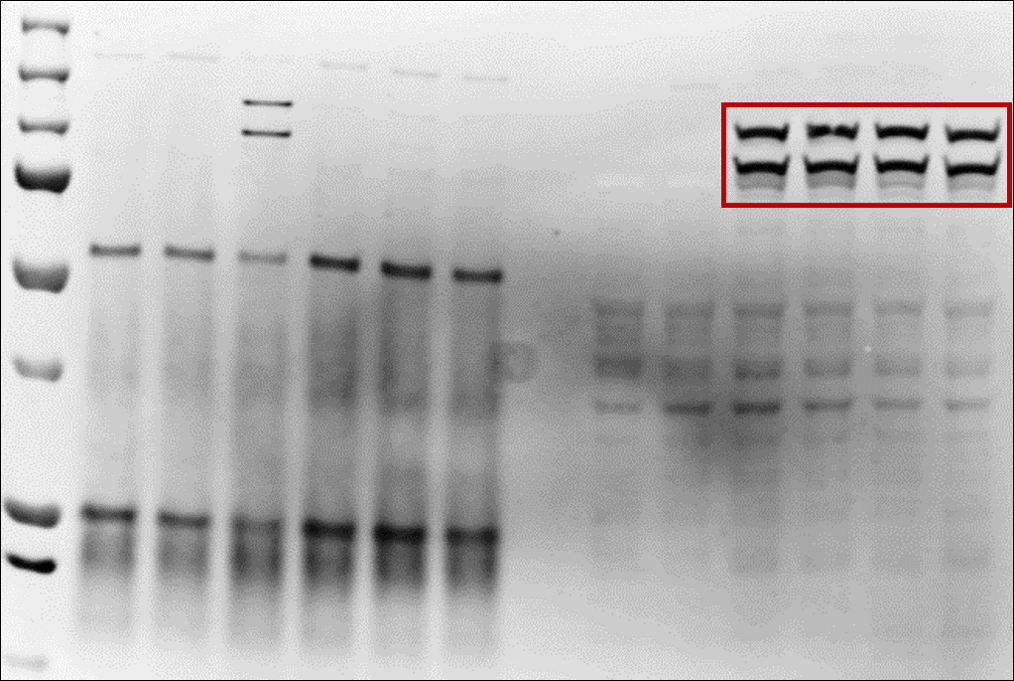

Supplement: Supplementary file 8 — Source data Fig. 3 [file 44319_2025_458_MOESM8_ESM.zip › C/3C_Highlighting_WCL_western blot_Myc.tif]

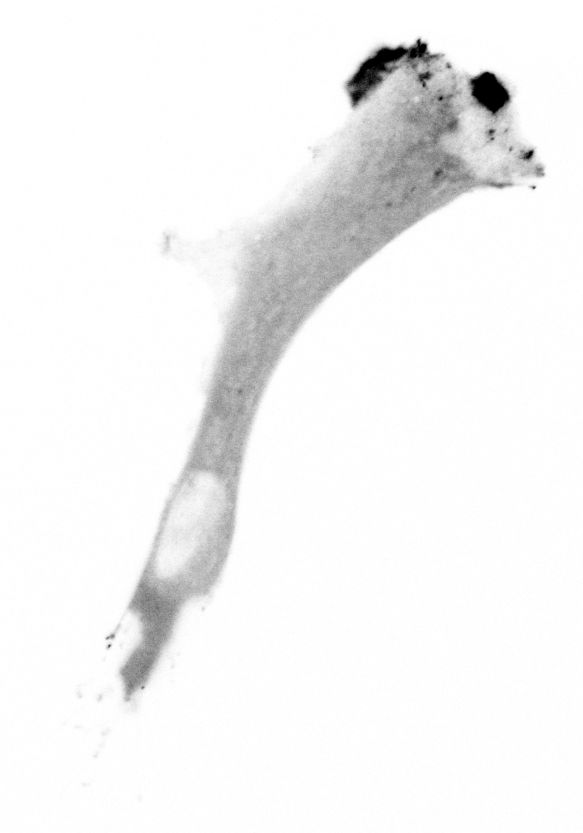

Supplement: Supplementary file 9 — Source data Fig. 4 [file 44319_2025_458_MOESM9_ESM.zip › 4A/4A_Hook3(VE)-FuRed_KIF1C(WT)-EGFP_EGFP.tif]

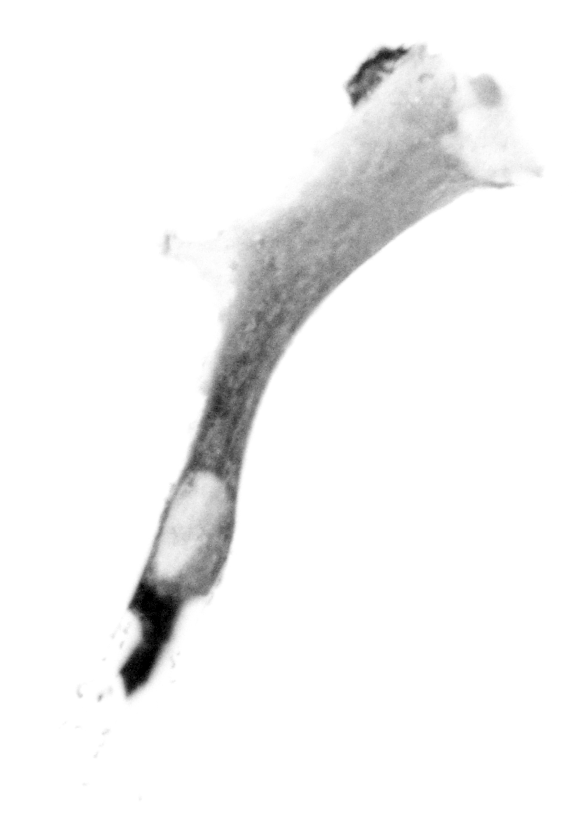

Supplement: Supplementary file 9 — Source data Fig. 4 [file 44319_2025_458_MOESM9_ESM.zip › 4A/4A_Hook3(VE)-FuRed_KIF1C(WT)-EGFP_FuRed.tif]

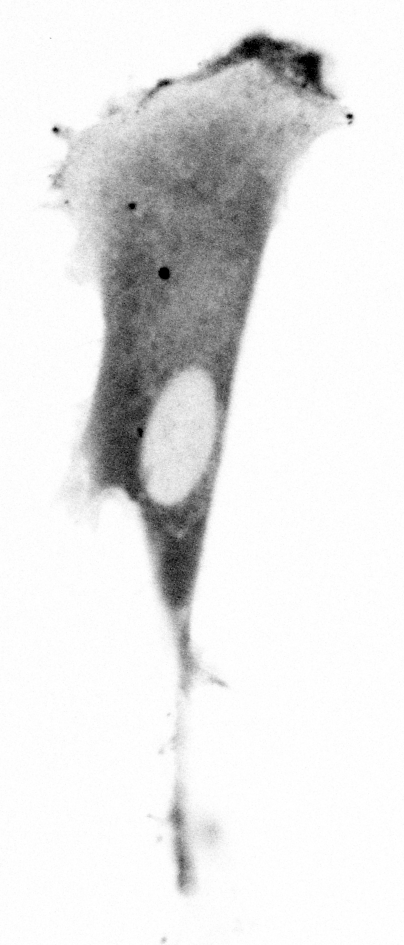

Supplement: Supplementary file 9 — Source data Fig. 4 [file 44319_2025_458_MOESM9_ESM.zip › 4A/4A_Hook3(VE)-FuRed_KIF1C(YAFA)-EGFP_EGFP.tif]

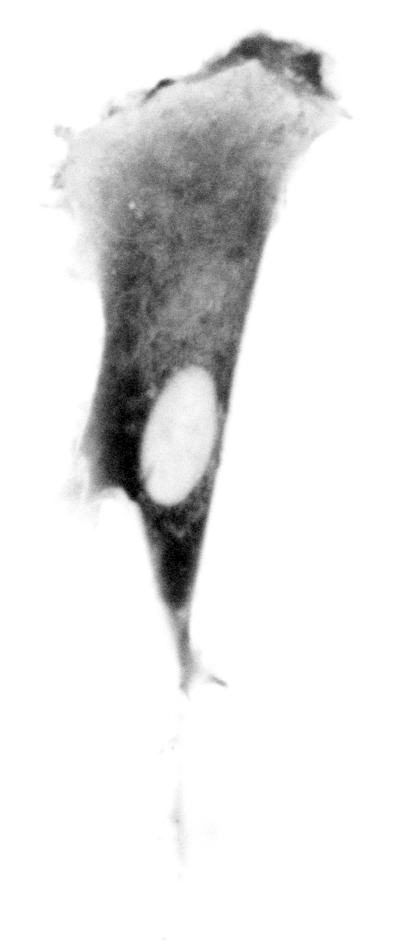

Supplement: Supplementary file 9 — Source data Fig. 4 [file 44319_2025_458_MOESM9_ESM.zip › 4A/4A_Hook3(VE)-FuRed_KIF1C(YAFA)-EGFP_FuRed.tif]

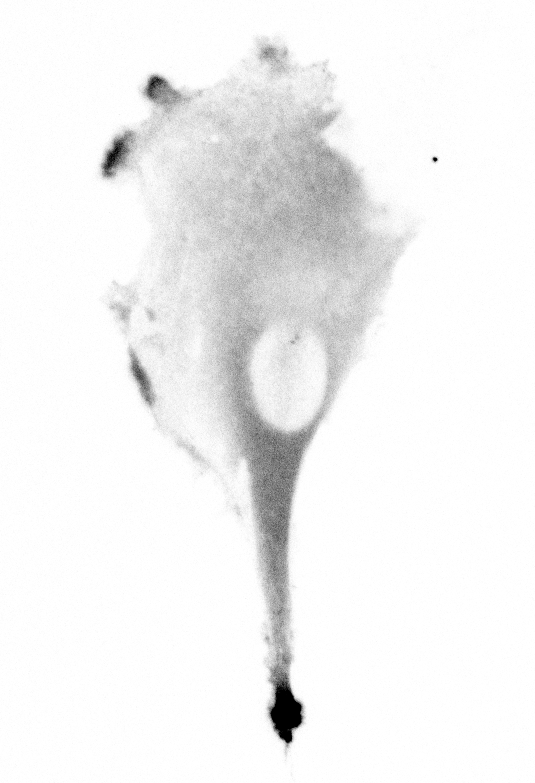

Supplement: Supplementary file 9 — Source data Fig. 4 [file 44319_2025_458_MOESM9_ESM.zip › 4A/4A_Hook3(WT)-FuRed_KIF1C(WT)-EGFP_EGFP.tif]

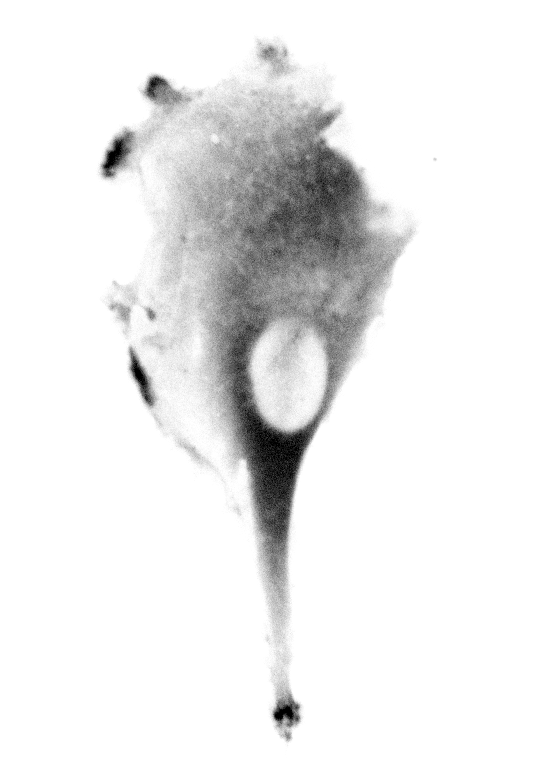

Supplement: Supplementary file 9 — Source data Fig. 4 [file 44319_2025_458_MOESM9_ESM.zip › 4A/4A_Hook3(WT)-FuRed_KIF1C(WT)-EGFP_FuRed.tif]

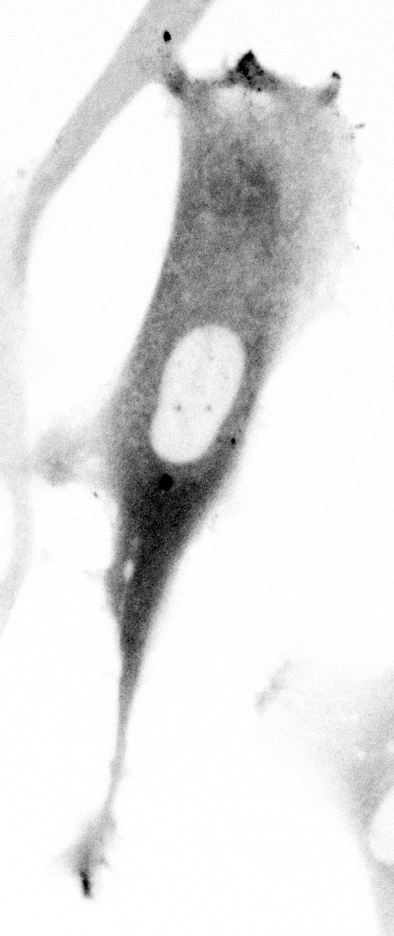

Supplement: Supplementary file 9 — Source data Fig. 4 [file 44319_2025_458_MOESM9_ESM.zip › 4A/4A_Hook3(WT)-FuRed_KIF1C(YAFA)-EGFP_EGFP.tif]

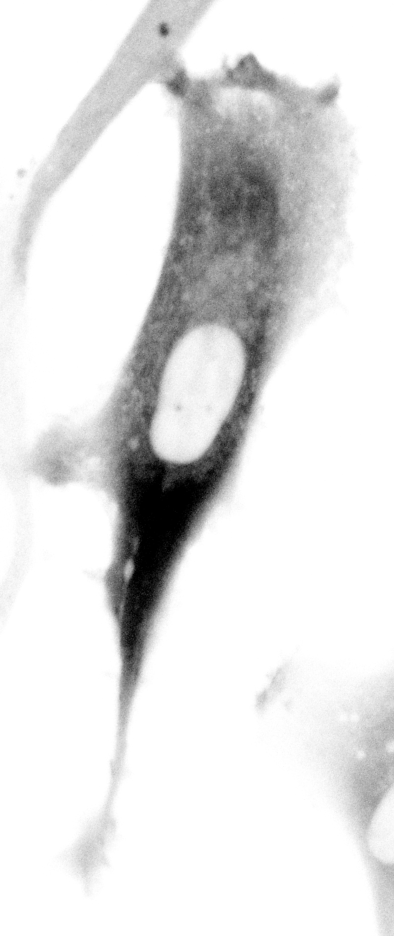

Supplement: Supplementary file 9 — Source data Fig. 4 [file 44319_2025_458_MOESM9_ESM.zip › 4A/4A_Hook3(WT)-FuRed_KIF1C(YAFA)-EGFP_FuRed.tif]

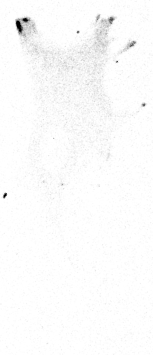

Supplement: Supplementary file 10 — Source data Fig. 5 [file 44319_2025_458_MOESM10_ESM.zip › 5B-C/1-1. 5B_Hook3(WT)_KIF1C(WT)_KIF1C-mTagBFP2.tif]

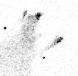

Supplement: Supplementary file 10 — Source data Fig. 5 [file 44319_2025_458_MOESM10_ESM.zip › 5B-C/1-1. 5C_Hook3(WT)_KIF1C(WT)_KIF1C-mTagBFP2_crop.tif]

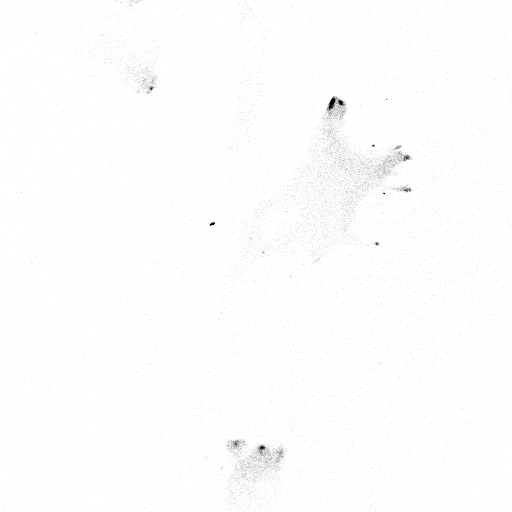

Supplement: Supplementary file 10 — Source data Fig. 5 [file 44319_2025_458_MOESM10_ESM.zip › 5B-C/1. Hook3(WT)_KIF1C(WT)_KIF1C-mTagBFP2_original.tif]

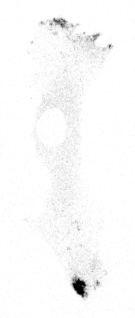

Supplement: Supplementary file 10 — Source data Fig. 5 [file 44319_2025_458_MOESM10_ESM.zip › 5B-C/10-1. 5B_Hook3(VE)_KIF1C(YAFA)_KIF1C-mTagBFP2.tif]

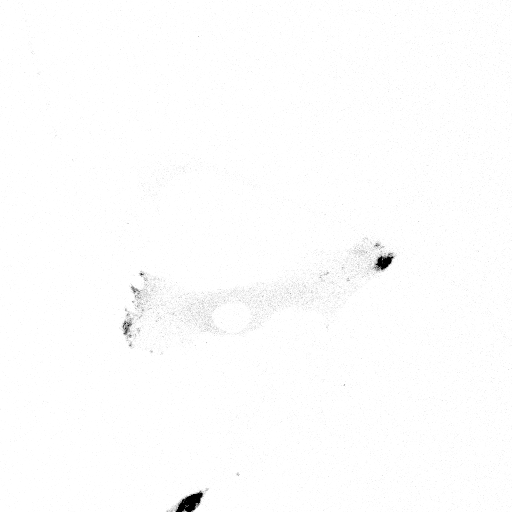

Supplement: Supplementary file 10 — Source data Fig. 5 [file 44319_2025_458_MOESM10_ESM.zip › 5B-C/10. Hook3(VE)_KIF1C(YAFA)_KIF1C-mTagBFP2.tif]

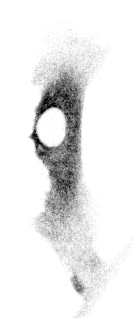

Supplement: Supplementary file 10 — Source data Fig. 5 [file 44319_2025_458_MOESM10_ESM.zip › 5B-C/11-1. 5B_Hook3(VE)_KIF1C(YAFA)_Hook3-FuRed-FRB.tif]

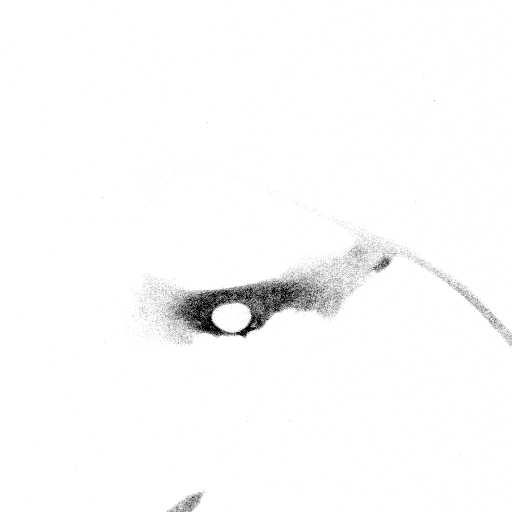

Supplement: Supplementary file 10 — Source data Fig. 5 [file 44319_2025_458_MOESM10_ESM.zip › 5B-C/11. Hook3(VE)_KIF1C(YAFA)_Hook3-FuRed-FRB_original.tif]

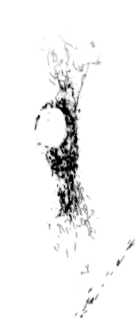

Supplement: Supplementary file 10 — Source data Fig. 5 [file 44319_2025_458_MOESM10_ESM.zip › 5B-C/12-1. 5B_Hook3(VE)_KIF1C(YAFA)_FKBP-EGFP-MoA.tif]

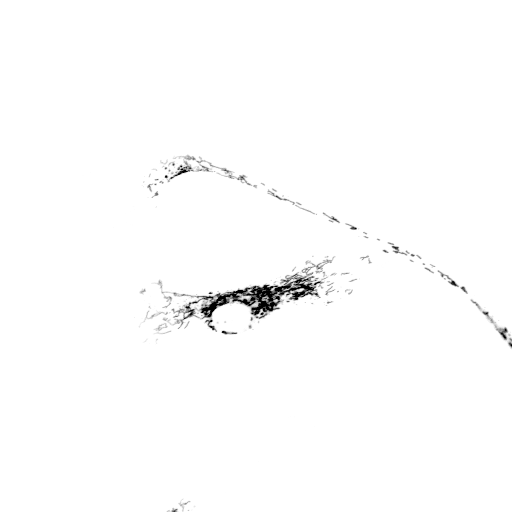

Supplement: Supplementary file 10 — Source data Fig. 5 [file 44319_2025_458_MOESM10_ESM.zip › 5B-C/12. Hook3(VE)_KIF1C(YAFA)_FKBP-EGFP-MoA_original.tif]

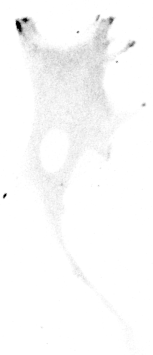

Supplement: Supplementary file 10 — Source data Fig. 5 [file 44319_2025_458_MOESM10_ESM.zip › 5B-C/2-1. 5B_Hook3(WT)_KIF1C(WT)_Hook3-FuRed-FRB.tif]

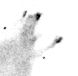

Supplement: Supplementary file 10 — Source data Fig. 5 [file 44319_2025_458_MOESM10_ESM.zip › 5B-C/2-2. 5C_Hook3(WT)_KIF1C(WT)_Hook3-FuRed-FRB_crop.tif]

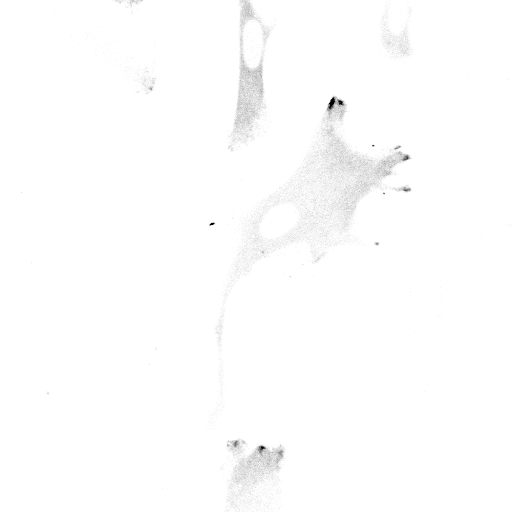

Supplement: Supplementary file 10 — Source data Fig. 5 [file 44319_2025_458_MOESM10_ESM.zip › 5B-C/2. Hook3(WT)_KIF1C(WT)_Hook3-FuRed-FRB_original.tif]

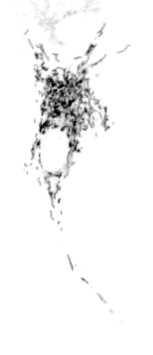

Supplement: Supplementary file 10 — Source data Fig. 5 [file 44319_2025_458_MOESM10_ESM.zip › 5B-C/3-1. 5B_Hook3(WT)_KIF1C(WT)_FKBP-EGFP-MoA.tif]

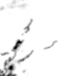

Supplement: Supplementary file 10 — Source data Fig. 5 [file 44319_2025_458_MOESM10_ESM.zip › 5B-C/3-2. 5C_Hook3(WT)_KIF1C(WT)_FKBP-EGFP-MoA_crop.tif]

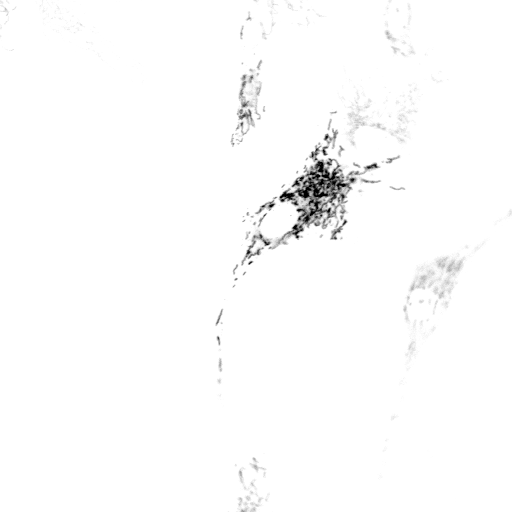

Supplement: Supplementary file 10 — Source data Fig. 5 [file 44319_2025_458_MOESM10_ESM.zip › 5B-C/3. Hook3(WT)_KIF1C(WT)_FKBP-EGFP-MoA_original.tif]

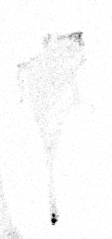

Supplement: Supplementary file 10 — Source data Fig. 5 [file 44319_2025_458_MOESM10_ESM.zip › 5B-C/4-1. 5B_Hook3(WT)_KIF1C(YAFA)_KIF1C-mTagBFP2.tif]

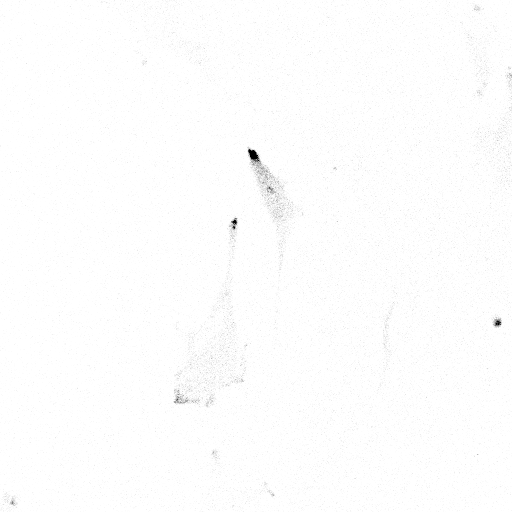

Supplement: Supplementary file 10 — Source data Fig. 5 [file 44319_2025_458_MOESM10_ESM.zip › 5B-C/4. Hook3(WT)_KIF1C(YAFA)_KIF1C-mTagBFP2_original.tif]

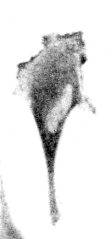

Supplement: Supplementary file 10 — Source data Fig. 5 [file 44319_2025_458_MOESM10_ESM.zip › 5B-C/5-1. 5B_Hook3(WT)_KIF1C(YAFA)_Hook3-FuRed-FRB.tif]

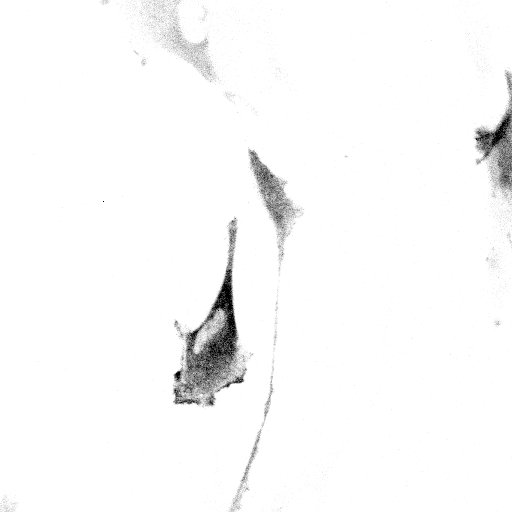

Supplement: Supplementary file 10 — Source data Fig. 5 [file 44319_2025_458_MOESM10_ESM.zip › 5B-C/5. Hook3(WT)_KIF1C(YAFA)_Hook3-FuRed-FRB_original.tif]

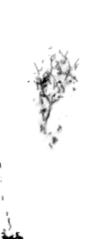

Supplement: Supplementary file 10 — Source data Fig. 5 [file 44319_2025_458_MOESM10_ESM.zip › 5B-C/6-1. 5B_Hook3(WT)_KIF1C(YAFA)_FKBP-EGFP-MoA.tif]

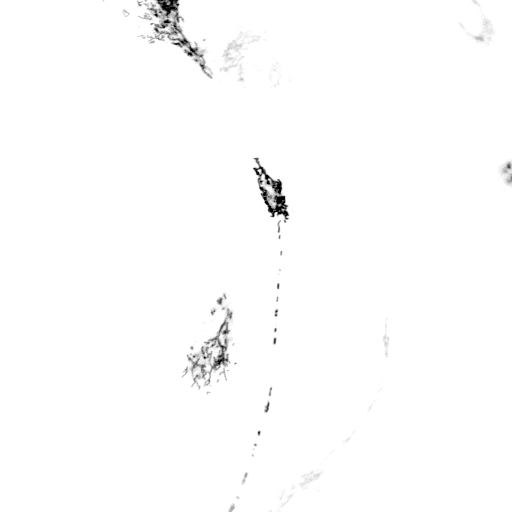

Supplement: Supplementary file 10 — Source data Fig. 5 [file 44319_2025_458_MOESM10_ESM.zip › 5B-C/6. Hook3(WT)_KIF1C(YAFA)_FKBP-EGFP-MoA_original.tif]

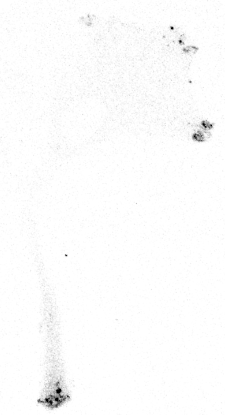

Supplement: Supplementary file 10 — Source data Fig. 5 [file 44319_2025_458_MOESM10_ESM.zip › 5B-C/7-1. 5B_Hook3(VE)_KIF1C(WT)_KIF1C-mTagBFP2.tif]

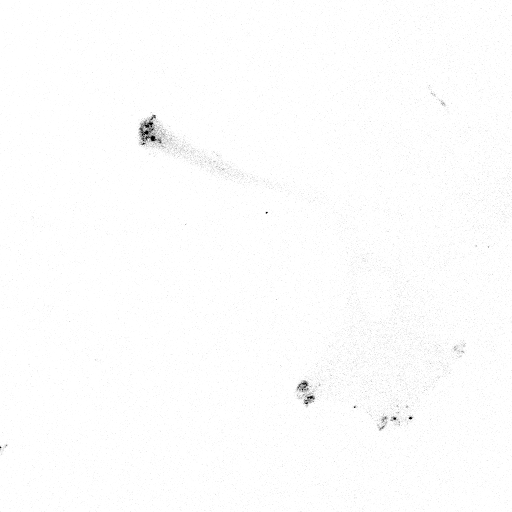

Supplement: Supplementary file 10 — Source data Fig. 5 [file 44319_2025_458_MOESM10_ESM.zip › 5B-C/7. Hook3(VE)_KIF1C(WT)_KIF1C-mTagBFP2_original.tif]

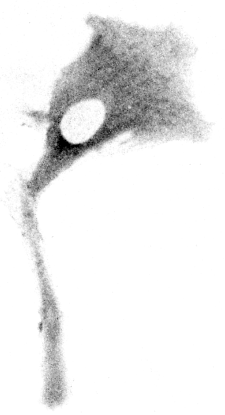

Supplement: Supplementary file 10 — Source data Fig. 5 [file 44319_2025_458_MOESM10_ESM.zip › 5B-C/8-1. 5B_Hook3(VE)_KIF1C(WT)_Hook3-FuRed-FRB.tif]

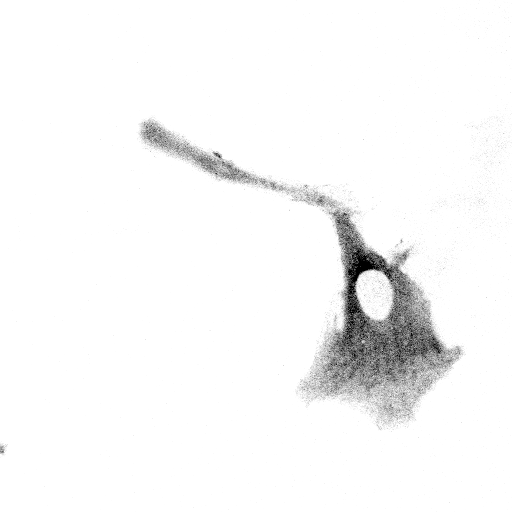

Supplement: Supplementary file 10 — Source data Fig. 5 [file 44319_2025_458_MOESM10_ESM.zip › 5B-C/8. Hook3(VE)_KIF1C(WT)_Hook3-FuRed-FRB_original.tif]

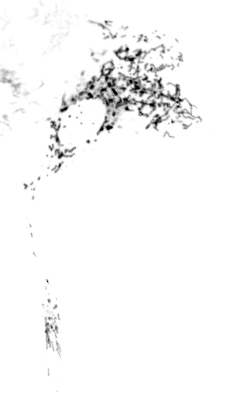

Supplement: Supplementary file 10 — Source data Fig. 5 [file 44319_2025_458_MOESM10_ESM.zip › 5B-C/9-1. 5B_Hook3(VE)_KIF1C(WT)_FKBP-EGFP-MoA.tif]

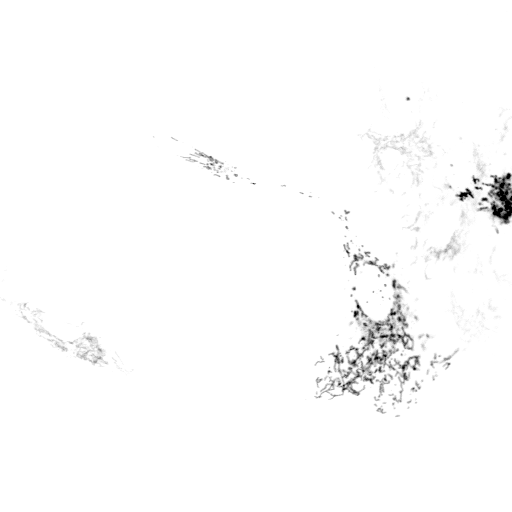

Supplement: Supplementary file 10 — Source data Fig. 5 [file 44319_2025_458_MOESM10_ESM.zip › 5B-C/9. Hook3(VE)_KIF1C(WT)_FKBP-EGFP-MoA_original.tif]
