# Supplementary material for: Molecular basis for assembly and activation of the Hook3 − KIF1C complex-dependent transport machinery
Source: EMBO Rep. 2025 May 1;26(11):2945–66. doi: 10.1038/s44319-025-00458-w (PMC12152161; doi:10.1038/s44319-025-00458-w)
Supplement: Supplementary file 11 — Expanded View Figures [file 44319_2025_458_MOESM11_ESM.pdf]

Expanded View Figures

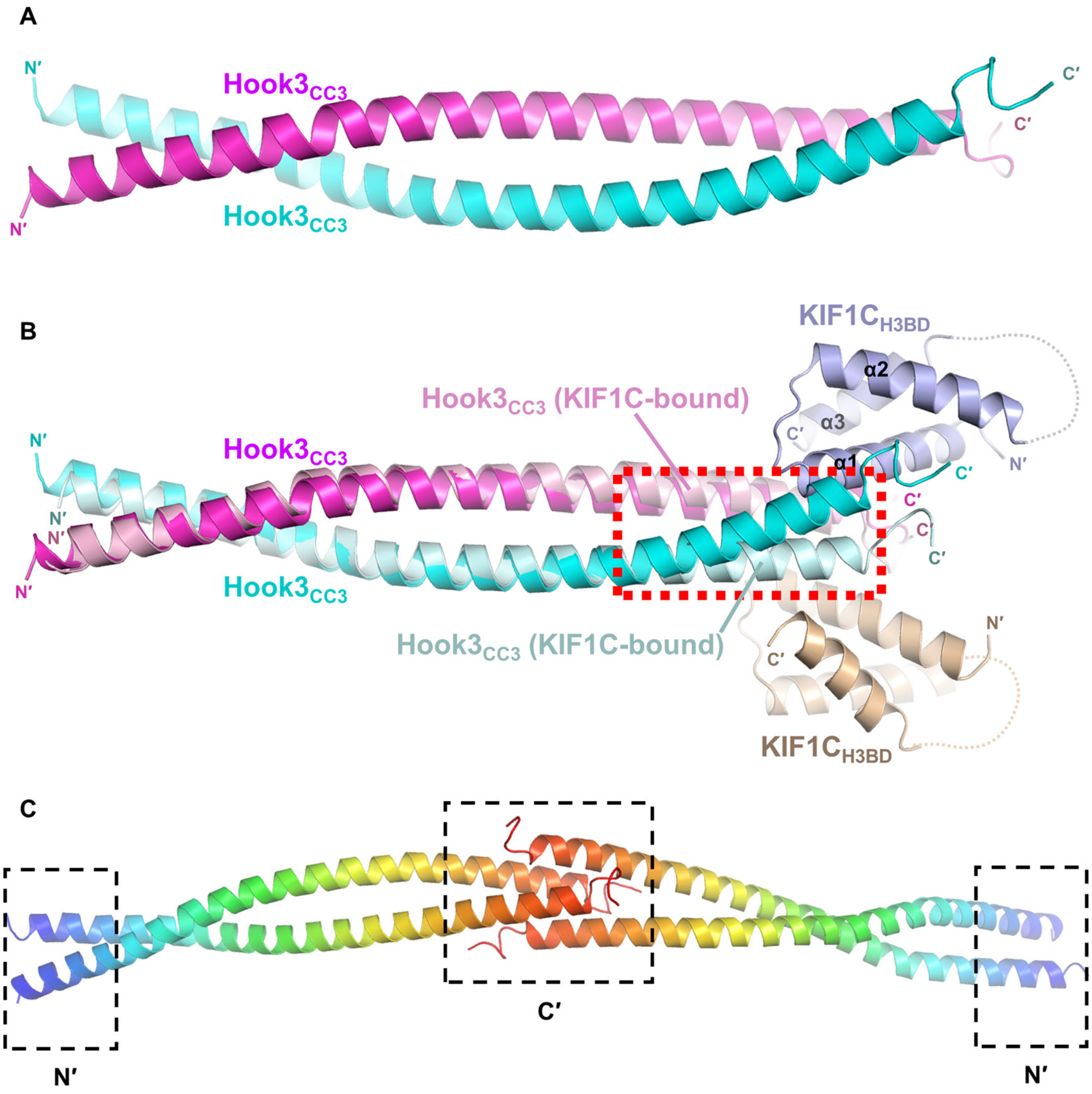

Figure EV1. Crystal structure of Hook3(553–624).

(**A**) Crystal structure of apo Hook3(553–624; magenta and cyan) shown as ribbon representation. The amino- and carboxyl-termini of each polypeptide are indicated as N′ and C′, respectively. (**B**) Cartoon representation of superposed structures of Hook3(553–624) in the apo form (magenta and cyan) and in KIF1C(714–809; navy and beige)-bound form (pink and mint). Dashed lines linking α2 and α3 of KIF1C represent invisible regions in the crystal structure owing to poor electron density. (**C**) Crystal packing of apo Hook3(553–624). Two symmetry mates of apo Hook3(553–624) are shown as ribbon representation. The four Hook3 molecules are gradually colored from blue (N′; residue 553) to orange (C′; residue 624).

 © The Author(s)

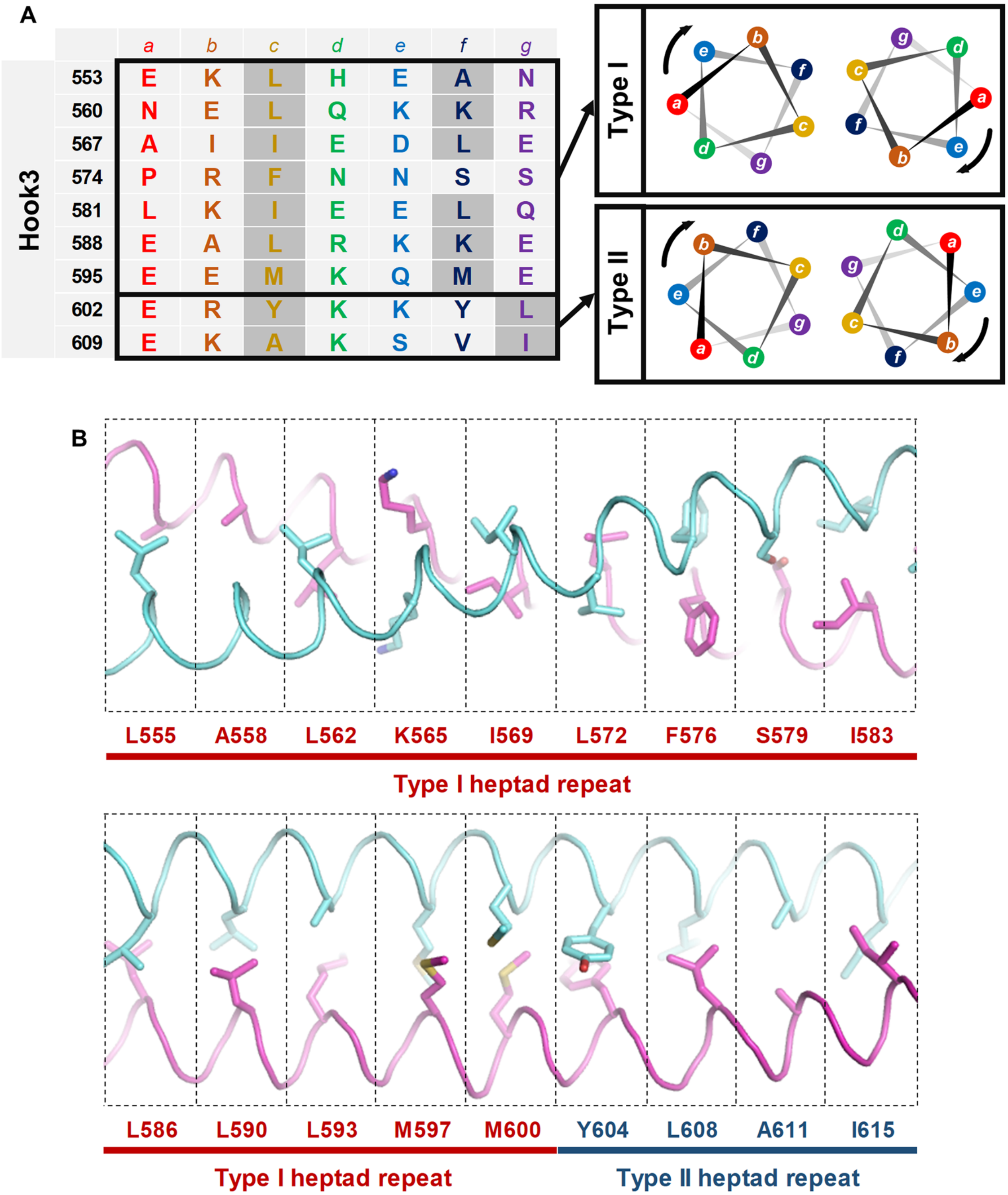

Figure EV2. Coiled-coil conformation of Hook3.

(**A**) Two heptad repeat types present in Hook3(553–624). (Left) Hook3 residues are assigned by the heptad repeat position (*a*–*g*). Hydrophobic residues involved in the intermolecular interaction for the coiled-coil formation are shaded in gray. (Right) Helical wheel representations. Black boxes indicate two different heptad repeat types in Hook3. (**B**) Dimeric interface of the Hook3 coiled coils is shown in pink and cyan. The key residues for the coiled-coil formation are represented as sticks with labels shown at the bottom (red, type I; blue, type II heptad repeat).

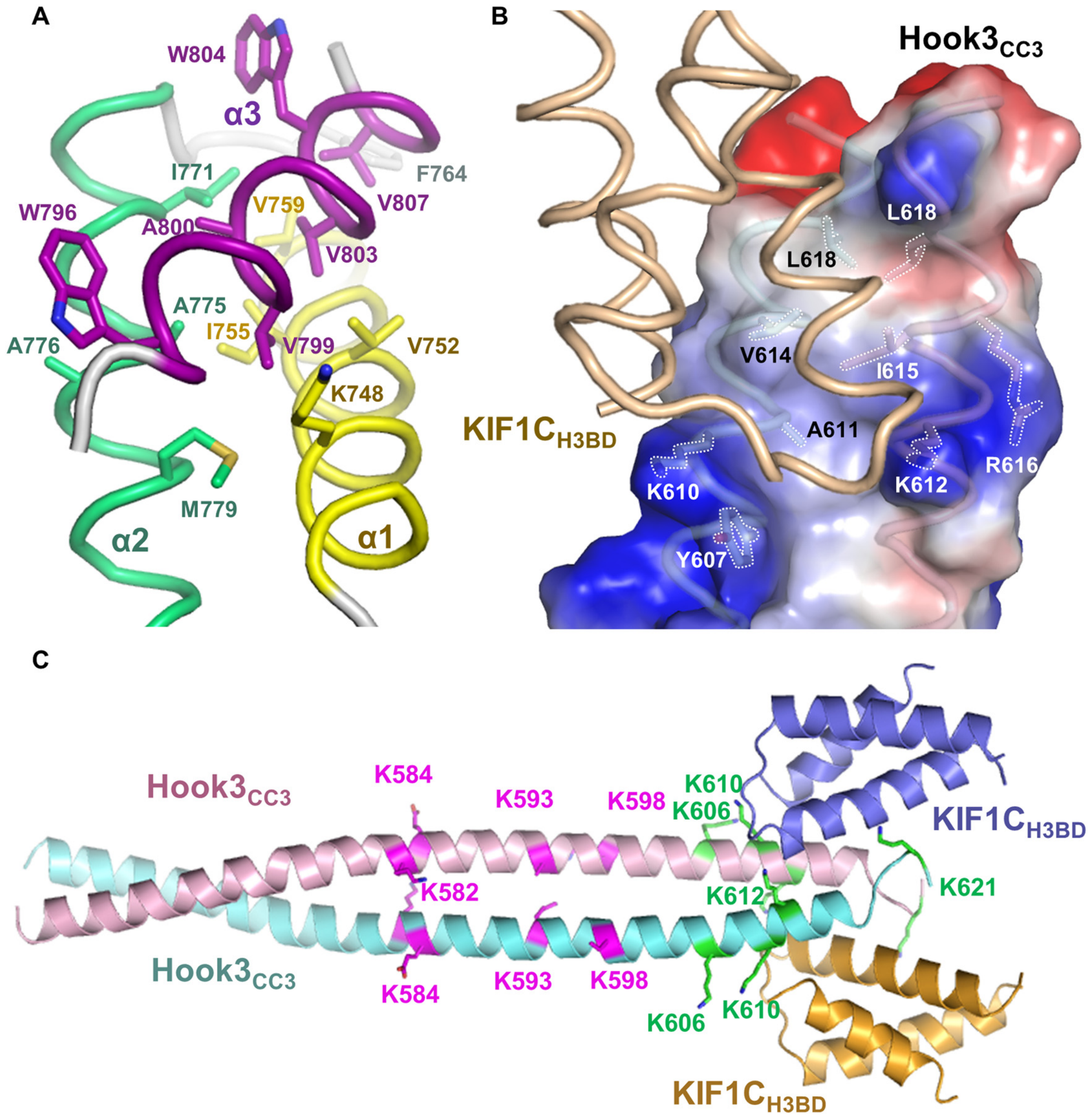

Figure EV3. Detailed structural analysis of the Hook3 – KIF1C complex.

(**A**) Intramolecular hydrophobic interaction in KIF1C(714–809). Loops are indicated in gray and the α1, α2, and α3 helices are represented in yellow, green, and purple, respectively. Hydrophobic residues involved in protein folding are represented as sticks and labeled. (**B**) Hook3(553–624) homodimer is shown as an electrostatic surface representation together with bound KIF1C(714–809) colored in beige. The Hook3 residues (black, from one Hook3 protomer; white, from another molecule) that constitute a large extended hydrophobic surface are shown in sticks with labels. (**C**) Lysine residues of Hook3 previously reported to be cross-linked with those of KIF1C (Abid Ali et al, 2025; Data ref: Abid Ali et al, 2025) are represented on our Hook3 – KIF1C complex structure as sticks with labels. Among them, those in the proximity of the complex interface are shown in green, whereas the rest are represented in magenta.

 © The Author(s)

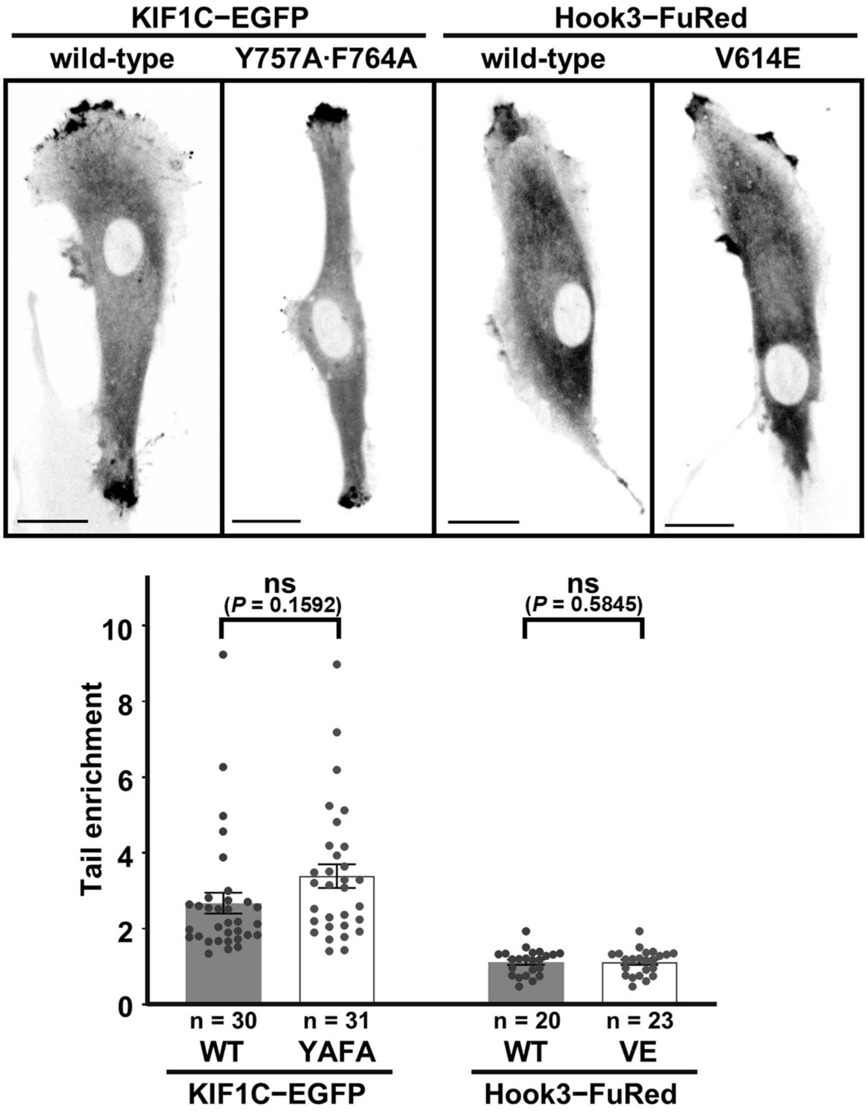

Figure EV4. Intracellular localization of Hook3 and KIF1C expressed separately.

(Top) Fluorescent images of RPE1 cells expressing KIF1C − EGFP or Hook3−FuRed at 16 h post transfection. Scale bars, 10 µm. (Bottom) Tail enrichment of KIF1C − EGFP and HooK3−FuRed in RPE1 cells. Tail/cytoplasm ratios of each protein were analyzed. Values are means ± standard error of the mean. ns, not significant by the Student's two-tailed *t* test. WT wild-type, YAFA Y757A · F764A, VE V614E.

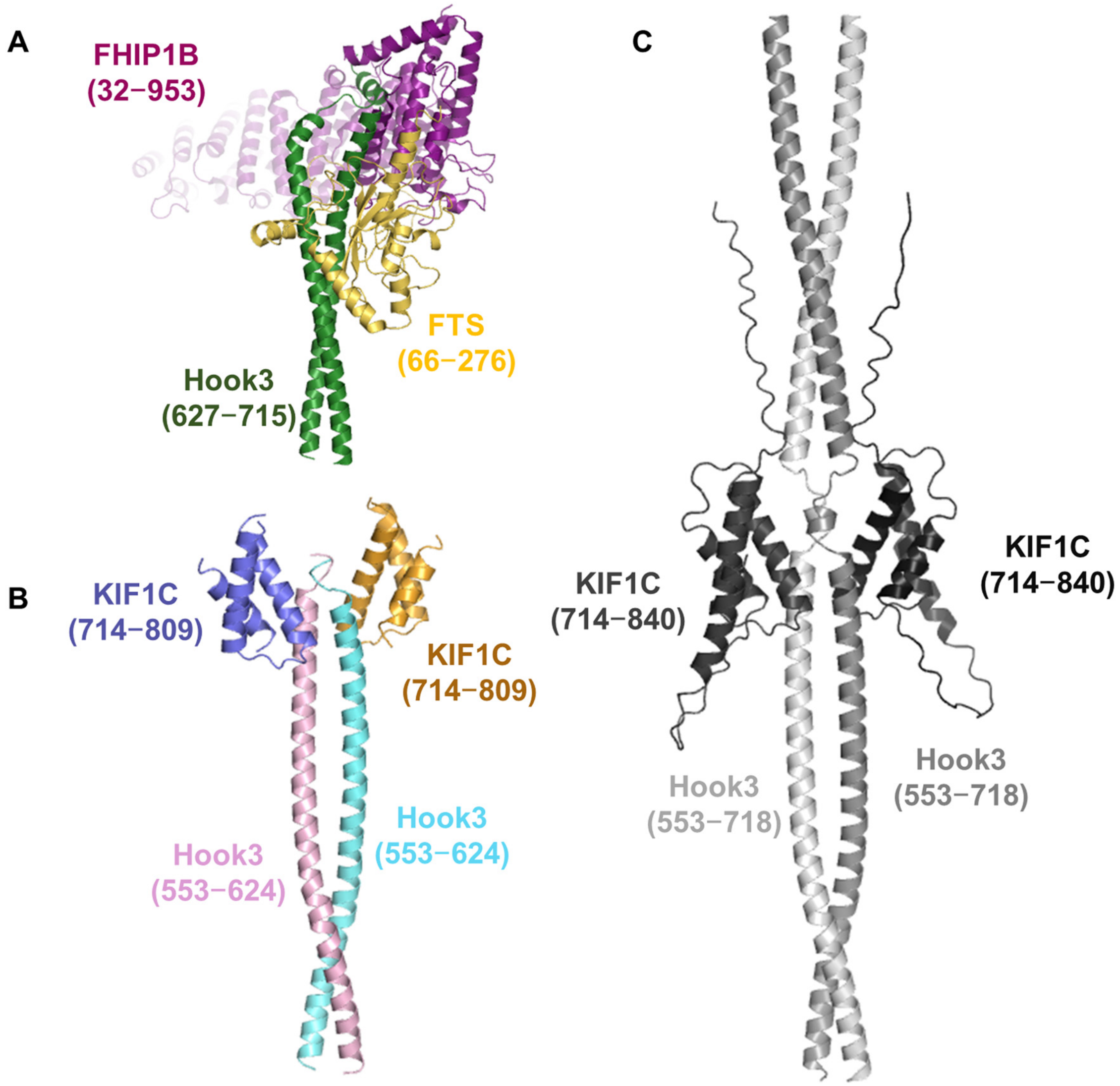

Figure EV5. Experimentally determined and AlphaFold2-predicted structures of Hook3-containing complexes.

(**A**) Cryo-EM structure of the FTS(66–276)–Hook3(627–715) – FHIP1B(32–953) complex (PDB code: 8QAT, Abid Ali et al, 2025; Data ref: Abid Ali et al, 2025). (**B**) Crystal structure of the Hook3(553–624) – KIF1C(714–809) complex (PDB code: 9KO8). (**C**) AlphaFold2-based structural model of the Hook3(553–718) – KIF1C(714–840) complex.

 © The Author(s)
